# Association of the single nucleotide polymorphisms in the extracellular matrix metalloprotease-9 gene with PACG in southern China

**Published:** 2009-07-24

**Authors:** Yanhong Cong, Xiangming Guo, Xing Liu, Dan Cao, Xiaoyun Jia, Xueshan Xiao, Shiqiang Li, Shaohua Fang, Qingjiong Zhang

**Affiliations:** State Key Laboratory of Ophthalmology, Zhongshan Ophthalmic Center, Sun Yat-Sen University, Guangzhou, People’s Republic of China

## Abstract

**Purpose:**

To study the association of the single nucleotide polymorphisms (SNPs) rs17576 and rs2250889 in the extracellular matrix metalloprotease-9 (*MMP-9*) gene with primary angle closure glaucoma (PACG) in southern Chinese patients.

**Methods:**

DNA samples were obtained from 211 adult patients with PACG and 205 control subjects to study the relationships between SNPs in *MMP-9* and PACG. Polymorphism analyses of rs17576 and rs2250889 in *MMP-9* were performed using the polymerase chain reaction,restriction fragment length polymorphism (RFLP), and direct DNA sequencing techniques. The association between genetic polymorphisms and the risk of PACG were estimated by χ2 test and logistic regression.

**Results:**

Genotyping of the *MMP-9* site (rs2250889) was significantly different between the two groups (P=0.004), and the odds ratio was OR=1.76 (95%CI: 1.264-2.449). The frequencies of the G/G genotype in the PACG and control groups were 9% and 2.9%, respectively. However, there were no significant differences between these two groups in the frequencies of the genotypes and alleles for rs17576 in *MMP-9*.

**Conclusion:**

The SNP rs2250889 located in *MMP-9* might be associated with PACG among southern Chinese people, and people with the G/G genotype are likely more susceptible to PACG. In contrast, the SNP rs17576 in *MMP-9* might not be related to PACG in the same population.

## Introduction

Glaucoma is the second leading cause of blindness worldwide. It is estimated to affect about 70 million people globally, of which about 6.7 million are bilaterally blind [1]. According to data on the epidemiology of glaucoma, primary angle closure glaucoma (PACG) is a major form of glaucoma in Asia [2], especially in populations of East Asian and Chinese descent [3-5], and the risk of PACG has been reported to increase with age [6,7].

PACG is characterized by partial or complete anterior chamber angle closure, which could lead to an increase in intraocular pressure (IOP), and thus damage to optic nerve. It has been suggested that a genetic link may be associated with the development of PACG in these populations [8]. The significant differences in incidence and prevalence among people from different regions are associated primarily with race, gender [9], and the conditions of anatomical structure [10,11]. With aging, the anterior chamber angles among Chinese people become increasingly narrow and the depths of their anterior chambers become steadily shallower. PACG patients are therefore usually found to have certain biometric ocular features, such as a shallow anterior chamber [12,13], increased thickness of the lens [14], and short axial length [15], often accompanied by hypermetropic refraction error [16].

Extracellular matrix remodeling is likely to be an important determinant for the short axial length in relatively small eyes. Therefore, it is logical that the enhanced activation of collagen degrading enzymes, particularly matrix metalloproteases (MMPs), might play a role in the remodeling process [17]. The 92 kDa subtype extracellular matrix metalloprotease-9 (MMP-9) [18], also known as 92 kDa gelatinase and type V collagenase could degrade type IV collagen, which is an important component of the extracellular matrix. It is expressed by the *MMP-9* gene with 13 exons, locating on 20q11.2-q13.1 [19], and it could be produced by normal mononuclear cells, granulocytes, smooth muscle cells, vascular endothelial cells, and so on. SNPs in *MMP-9* might cause a change in function of MMP-9 and thus affect extracellular matrix remodeling. A recent study showed that rs17576 is associated with susceptibility to acute PACG among Taiwanese patients [20], however, no such association was found in a similar study focused on Singaporean patients [21].

This research was a case-control study that considered the southern Chinese population and aimed to determine whether the single nucleotide polymorphisms rs17576 and rs2250889 in *MMP-9* were related to PACG.

## Methods

### Subjects

All of the patients were recruited from the clinics at Ophthalmic Center at Sun Yat-sen University (Guangzhou, China). The patients were from the native ethnic Han Chinese population of southern China. Informed consent conforming to the tenets of the Declaration of Helsinki was obtained from each participant prior to the study. Medical and ophthalmic histories were obtained. Ophthalmological examinations, including visual acuity, slit lamp biomicroscopy, anterior chamber depth, intraocular pressure (IOP) measurement, and ophthalmoscopic observation were performed by Dr. Liu of Zhongshan Ophthalmic Center at Sun Yat-sen University. A total of 211 patients collected in the PACG group met the following diagnostic criteria: (1) chamber angle closure of at least 180 degrees; (2) IOP level over 21 mmHg using Goldmann applanation tonometry; (3) typical glaucomatous visual defect by visual field examination; (4) typical glaucomatous optic nerve injury with a C/D ratio over 0.5 by funduscope examination; and (5) secondary glaucoma was excluded. In the control group, a total of 205 native southern Han Chinese individuals with an age range from 55 to 75 years old were enrolled into the control group according to the following criteria: (1) no symptoms or signs of PACG mentioned above; (2) glaucoma history and familial glaucoma history were excluded; and (3) with no ophthalmic diseases besides cataract.

### DNA extraction, amplification and SNP site assessment

**Figure 1:**
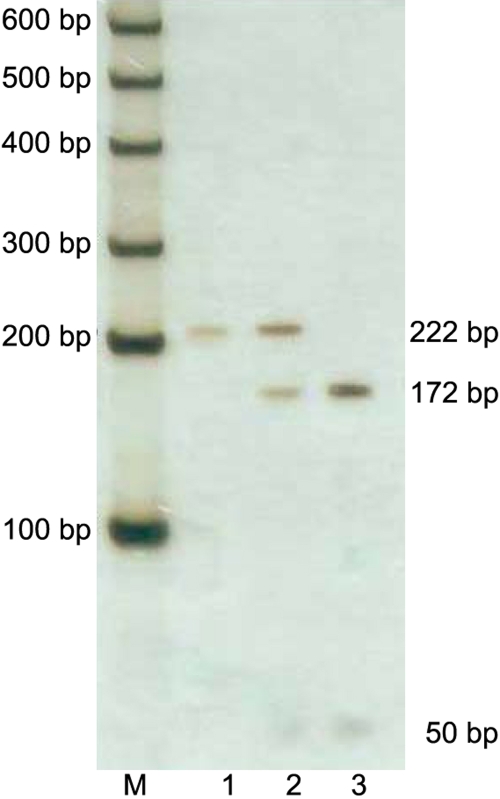
The result of restriction enzyme digestion for rs17567. The PCR products containing rs17576 were digested by restriction enzyme Bsob1. Fragment with the G allele in site rs17576 could be cut into two periods. Lane M stands for the lane of the Marker of 100 bp to 600 bp with intervals of 100 bp ladder; Lane 1 stands for genotype A/A (222 bp); Lane 2 stands for genotype A/G (222 bp, 172 bp, and 50bp); Lane 3 stands for genotype G/G (172 bp and 50 bp).

Genomic DNA was prepared from peripheral venous blood leucocytes. Two pairs of primers (Table 1) designed with Primer Premier 5 were used to amplify *MMP-9* sequences containing SNPs rs17576 and rs2250889 (NC_000020.9). The PCR programs used comprised an initial denaturation at 95 °C for 5 min followed by 35 cycles of 95 °C for 30 s, 64 °C/64.2 °C for 30 s, and 72 °C for 30 s. After the last cycle, the reaction was held at 72 °C for 5 min. The PCR products were electrophoresed on an agarose gel (1.5%) to confirm the correct amplicon size. Restriction enzyme digestion was performed on rs17576 PCR products using the restriction enzyme Bsob1 (New England Biolabs, Beijing, China) following the supplier’s protocol. After digestion, all fragments were resolved on polyacrylamide gel (8% PAG, 49:1, 1X TBE) at 15 W for 1 h. Subsequently, the gel was silver stained the same as for the SSCP analysis described previously [22]. A single fragment of 211 base pairs (bp) was identified as homozygous (A/A), three fragments of 222, 172, and 50 bp were identified as heterozygous (A/G), and two fragments of 172 and 50 bp were identified as the homozygous (G/G) genotype (Figure 1). Patient and control samples containing rs2250889 in *MMP-9* were sequenced with the ABI BigDye Terminator Cycle Sequencing kit v3.1 (ABI Applied Biosystems, Foster City, CA) [23], using an ABI 377 or 3100 sequencer (Applied Biosystems). The fragment containing rs2250889 was amplified and sequenced by the forward primer set. Comparative sequencing analysis between these samples and the standard sequence (NC_000020.9) was performed using SeqMan II (DNAStar Inc., Madison, WI).

**Table 1:** Primers for each PCR product containing rs17576 and rs2250889.

**Name**	**SNP**	**Primer**	**Product length (bp)**	**Primer sequence (5’→3’)**
rs17576	A/G	Forward		CAATTCACCCTCCCGCACTC
		Reverse	222	GGAAGATGAATGGAAACTGG
rs2250889	C/G	Forward		GTATTTGTTCAAGGATGGGTG
		Reverse	367	AGACGTTTCGTGGGTTAT

Forward and reverse primers were designed with Primer Primier 5 to amplify the fragments containing rs17576 and rs2250889 in *MMP9*. Primer sequences, sizes of PCR products and the annealing temperature used for the amplification are listed above.

### Statistical analysis

All detected SNPs were assessed for Hardy-Weinberg disequilibrium using the χ^2^ test. In addition, the χ^2^ test was used to determine the differences in the observed genotype and allele frequencies between the two groups. These statistical analyses were all performed using SPSS 13.0. After Bonferroni correction, a p-value < 0.025 was considered significant (α= 0.05/2 = 0.025).

## Results

This research considered 211 PACG patients with a mean age of 64.8±9.8 (69.2% females and 30.8% males), and 205 controls with a mean age of 68.0±7.2 (65.8% females and 34.2% males). The data indicated a higher risk of PACG among females in southern China, a finding that was confirmed in a previous report.

**Figure 2:**
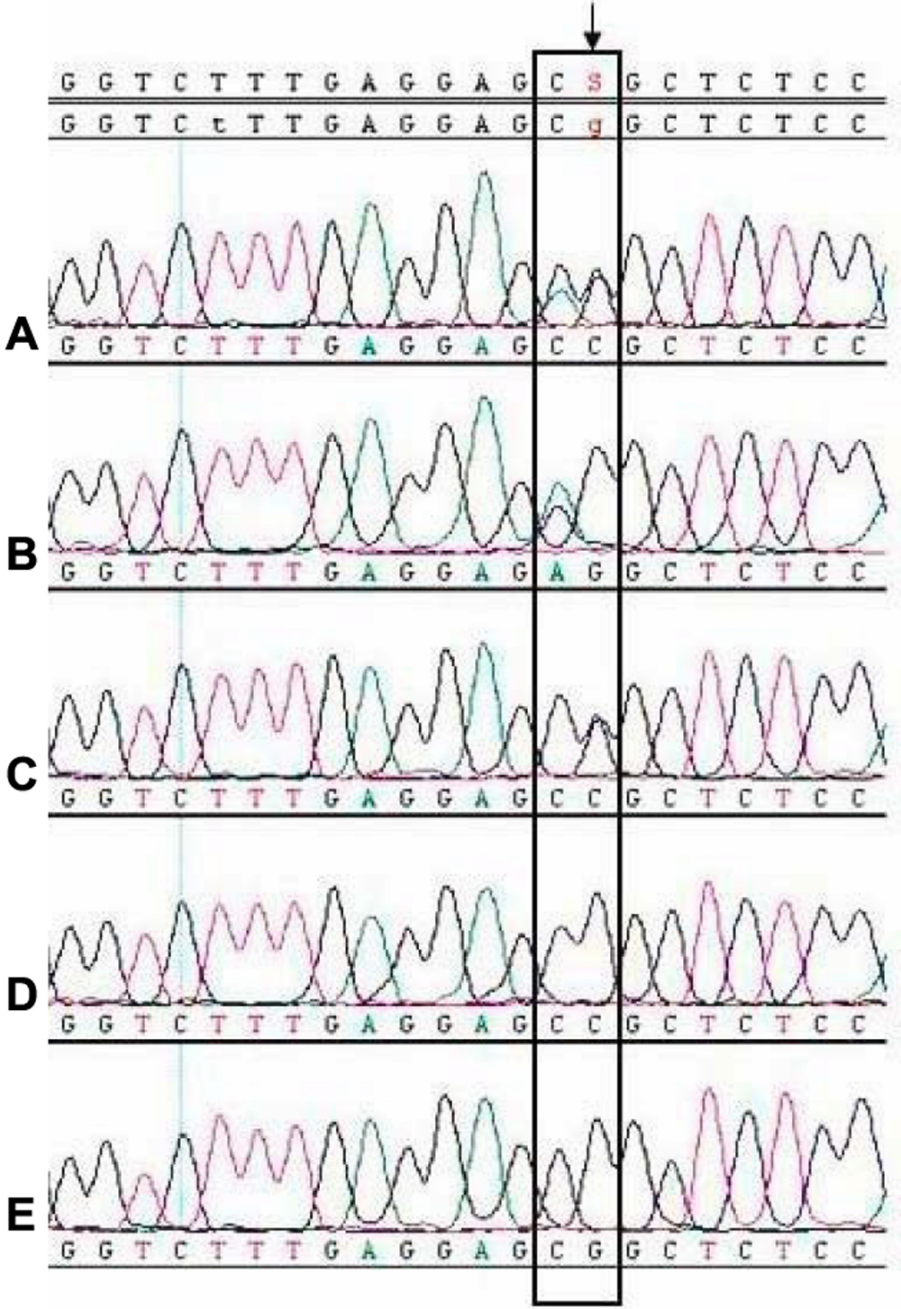
Five genotypes of Direct sequencing map for rs2250889 in MMP9 are shown. The single base directed with a black arrowhead was the SNP site rs2250889. The two bases collected in the black frame were two single nucleotide polymorphisms. The single base before site rs2250889 was a new SNP found in our research, and it was a linkage mutation, which did not change amino acid coding. Genotypes of rs2250889 in **A**, **B**, **C**, **D**, and **E** were separately C/G, G/G, C/G, C/C, G/G, while genotypes of the single base before it in **A**, **B**, **C**, **D**, and **E** were separately A/C, A/C, C/C, C/C, C/C.

The frequencies of rs17576 and rs2250889 genotypes and alleles in the PACG and control subjects conformed to the Hardy-Weinberg equilibrium. The genotype and allele frequencies of rs17576 and rs2250889 in the PACG and control groups are shown in Table 2. The homozygous A/A genotype frequency of rs17576 was 7.6% in the PACG group and 2.9% in control group; however, no significant difference was found in the genotype and alleles frequencies between the PACG and control groups (χ^2^=4.57, p=0.102). According to the sequencing result, there were five types of genotype in fragments containing rs2250889 (Figure 2). The homozygous G/G, heterozygous G/C and homozygous C/C genotypes of rs2250889 were 9.0%, 54.0%, and 37.0%, respectively, in the PACG group and 2.9%, 67.3% and 39.8%, respectively, in the control group. A significant difference in the rs2250889 genotype frequencies was found between the PACG and control groups (χ^2^=11.04, p=0.004). A linkage mutation of a single base (c.1720C>A) in 9 out of 211 subjects in the PACG group was also detected. The linkage mutation was located just before the site of SNP rs2250889 (c.1721C>G). When the allele at the site of rs2250889 was G, the base just before it might change from C to A. None of these mutations were observed among the subjects in the control group. This mutation did not change the amino acid encoding, and it was a new SNP that had not been reported previously.

**Table 2:** Sequence alterations detected and investigated in PACG and control groups.

**Sequence change**		**Allele distribution (%)**		**Allele** **association** **p-value**	**Odds ratio** **(95%CI)**		**Genotype distribution (%)**		**Genotype** **association** **p-value**
		**PACG** **n=211**	**Control** **n=205**				**PACG** **n=211**	**Control** **n=205**	
rs17576	A	108(25.6)	87(21.2)	0.126	1.28 (0.95-1.76)	AA	16 (7.6)	6 (2.9)	0.102
c.836A>G; p.279Q>R	G	314(74.4)	323(78.8)			AG	76 (36.0)	75 (36.6)	
						GG	119 (56.4)	124 (60.5)	
rs2250889	G	116(27.5)	73(17.8)	0.001	1.75 (1.26-2.44)	GG	19 (9.0)	6 (2.9)	0.004
c.1722G>C; p.574R>P	C	306(72.5)	337(82.2)			GC	78 (37.0)	61 (29.8)	
						CC	114 (54.0)	138 (67.3)	

Listed are allele and genotype frequencies of SNP rs17576 and rs2250889 in *MMP9* among PACG and control groups from the south of Chinese population. The frequencies of genotypes distributions in PACG and control subjects conformed to the Hardy-Weinberg equilibrium. Data are given as numbers (percentage). The p-value was calculated using the χ2 test (After Boferrni correction, a p <0.025 was considered to be significant). PACG indicates primary angle-closure glaucoma.

## Discussion

Primary angle-closure glaucoma (PACG) is a major cause of visual morbidity in East Asia and China, and it is expected to become an even more serious problem as world population and longevity increases. Racial differences in the prevalence of PACG and the familial tendency towards the disease suggest a genetic basis for the condition. Our study is the first to report an association of the SNP rs2250889 in *MMP-9* with PACG. Further, it is the first in China to discuss the association of rs17576 in *MMP-9* with PACG among the native southern Chinese population. Through a case-control study, we found a significant association between rs2250889 in *MMP-9* and PACG (p=0.004). This finding suggests that rs2250889 in *MMP-9* might be a risk factor for PACG in the southern Chinese population.

*MMP-9* is located in chromosome 20q11.2-q13.1 and contains 13 exons. Some research has shown that using ELISA, in both pro and active forms of MMP-9 (GLB) bind to type-I and type-IV collagens, gelatin and laminin [24]. Matrix metalloprotease-9 cleaves type IV collagen and is important for the remodeling of the ECM. Liu et al [25]. found a decreased mRNA transcription of *MMP-9* in the Tenon’s capsule in PACG. Altered enzyme activities might be attributable to variants in or near this gene. For example, variants located in the promoter region of a gene predominantly affect the transcriptional activity and therefore the gene expression, while an amino acid change of nonsynonymous SNPs in the coding region could also affect the protein’s tertiary structure and thus its biophysical and biochemical activity. SNPs rs17576 and rs2250889 are nonsynonymous single base changes in coding regions, and their change might play a role in the structure and function of protein MMP-9. Located in the second COOH-terminal hemopexin-like domain of *MMP-9*, rs2250889 (c.1721C>G, p.574P>R) represents a substantial change since Pro is a cyclic, nonpolar amino acid while Arg is basic and has a positive charge [26]. SNP in *MMP-9* rs17576 (c.836G>A, p.279Q>R) is a nonconservative amino acid substitution that resides within the highly conserved gelatinase-specific fibronectin type II domain (FN2) [24], which presumably enhances substrate binding. These residues might have significant interactions with surrounding residues, so variations in these amino acids could affect protein stability and function.

Recently, the association of SNPs in potential genes (*MMP-9*) with PACG has been studied by several researchers in Taiwan and other nations. Taiwanese researchers suggested an association of SNP rs17576 with acute PACG in a case-control study comprised of 78 patients and 86 controls. Singaporean researchers found no association of rs17576 with PACG among 217 PACG patients (85 acute PACG and 132 chronic PACG) and 83 controls. The results of the association study between SNPs in genes with diseases would be affected greatly by the number of subjects and ethnic distribution. In our study, all subjects were from the native southern Han Chinese population. Further, the number of subjects in the PACG and control groups were above 200, and subjects in the two groups were age and gender-matched. The data indicated a significant association of rs2250889 with susceptibility to PACG, but the exact mechanism by which it affected the function of MMP-9 was not known. The patients in our research have regular axial length and no obvious microphthalmia. Although our results suggested an association of this SNP with PACG, we were uncertain whether this SNP was related determinately to PACG. Our results did not support a significant association of rs17576 with PACG, but the frequency of the A/A genotype was much higher in the PACG group than in the control group.

## References

[1] Quigley HA Number of people with glaucoma worldwide. Br J Ophthalmol 1996 80 389 93 8695555 10.1136/bjo.80.5.389 PMC505485

[2] Roedl JB Bleich S Schlotzer-Schrehardt U von Ahsen N Kornhuber J Naumann GO Kruse FE Junemann AG Increased homocysteine levels in tear fluid of patients with primary open-angle glaucoma. Ophthalmic Res 2008 40 249 56 18437035 10.1159/000127832

[3] Congdon N Wang F Tielsch JM Issues in the epidemiology and population-based screening of primary angle-closure glaucoma. Surv Ophthalmol 1992 36 411 23 1589856 10.1016/s0039-6257(05)80022-0

[4] Foster PJBJ Alsbirk PH Munkhbayar D Uranchimeg D Johnson GJ Glaucoma in Mongolia-a population-based survey in Hovsgol Province, Northern Mongolia. Arch Ophthalmol 1996 114 1235 41 8859083 10.1001/archopht.1996.01100140435011

[5] Foster PJ Oen FT Machin D Ng TP Devereux JG Johnson GJ Khaw PT Seah SK The prevalence of glaucoma in Chinese residents of Singapore: a cross-sectional population survey of the Tanjong Pagar district. Arch Ophthalmol 2000 118 1105 11 10922206 10.1001/archopht.118.8.1105

[6] Yamamoto T Iwase A Araie M Suzuki Y Abe H Shirato S Kuwayama Y Mishima HK Shimizu H Tomita G Inoue Y Kitazawa Y The Tajimi Study report 2: prevalence of primary angle closure and secondary glaucoma in a Japanese population. Ophthalmology 2005 112 1661 9 16111758 10.1016/j.ophtha.2005.05.012

[7] He M Foster PJ Ge J Huang W Zheng Y Friedman DS Lee PS Khaw PT Prevalence and clinical characteristics of glaucoma in adult Chinese: a population-based study in Liwan district, Guangzhou. Invest Ophthalmol Vis Sci 2006 47 2782 8 16799014 10.1167/iovs.06-0051

[8] Dai X Nie S Ke T Liu J Wang Q Liu M [Two variants in MYOC and CYP1B1 genes in a Chinese family with primary angle-closure glaucoma]. Zhonghua Yi Xue Yi Chuan Xue Za Zhi. 2008 25 493 6 18841557

[9] Bonomi L Marchini G Marraffa M Bernardi P De Franco I Perfetti S Varotto A Epidemiology of angle-closure glaucoma: prevalence, clinical types, and association with peripheral anterior chamber depth in the Egna-Neumarket Glaucoma Study. Ophthalmology 2000 107 998 1004 10811096 10.1016/s0161-6420(00)00022-1

[10] David R Tessler Z Yassur Y Epidemiology of acute angle-closure glaucoma: incidence and seasonal variations. Ophthalmologica 1985 191 4 7 4034165 10.1159/000309530

[11] Buhrmann RR Quigley HA Barron Y West SK Oliva MS Mmbaga BB Prevalence of glaucoma in a rural East African population. Invest Ophthalmol Vis Sci 2000 41 40 8 10634599

[12] Zhuang X Zhu RR Guan HJ Huang CH Shi WP Jiang SY [A case-control study of factors associated with primary angle-closure glaucoma]. Zhonghua Yan Ke Za Zhi 2008 44 503 6 19035239

[13] Lin YW Wang TH Hung PT Biometric study of acute primary angle-closure glaucoma. J Formos Med Assoc 1997 96 908 12 9409125

[14] Foster PJBJ Alsbirk PH Munkhbayar D Uranchimeg D Johnson GJ Glaucoma in Mongolia-a populatoin-based survey in Hovsgol Province, Northern Mongolia. Arch Ophthalmol 1996 114 1235 41 8859083 10.1001/archopht.1996.01100140435011

[15] Abu-Amero KK Morales J Osman MN Bosley TM Nuclear and mitochondrial analysis of patients with primary angle-closure glaucoma. Invest Ophthalmol Vis Sci 2007 48 5591 6 18055808 10.1167/iovs.07-0780

[16] Congdon NG Quigley HA Hung PT Wang TH Ho TC Screening techniques for angle-closure glaucoma in rural Taiwan. Acta Ophthalmol Scand 1996 74 113 9 8739673 10.1111/j.1600-0420.1996.tb00053.x

[17] McBrien NAGA TIMP-2 regulation of MMP-2 activity during visually guided remodeling of the tree shrew sclera in lens-induced myopia. Invest Ophthalmol Vis Sci 2001 42 Suppl 314

[18] Nagase H Barrett AJ Woessner JF Jr Nomenclature and glossary of the matrix metalloproteinases. Matrix Suppl 1992 1 421 4 1480083

[19] St Jean PL Zhang XC Hart BK Lamlum H Webster MW Steed DL Henney AM Ferrell RE Characterization of a dinucleotide repeat in the 92 kDa type IV collagenase gene (CLG4B), localization of CLG4B to chromosome 20 and the role of CLG4B in aortic aneurysmal disease. Ann Hum Genet 1995 59 17 24 7762981 10.1111/j.1469-1809.1995.tb01602.x

[20] Wang IJ Chiang TH Shih YF Lu SC Lin LL Shieh JW Wang TH Samples JR Hung PT The association of single nucleotide polymorphisms in the MMP-9 genes with susceptibility to acute primary angle closure glaucoma in Taiwanese patients. Mol Vis 2006 12 1223 32 17110919

[21] Aung T Yong VH Lim MC Venkataraman D Toh JY Chew PT Vithana EN Lack of association between the rs2664538 polymorphism in the MMP-9 gene and primary angle closure glaucoma in Singaporean subjects. J Glaucoma 2008 17 257 8 18552608 10.1097/IJG.0b013e31815c3aa5

[22] Zhang Q Minoda K Detection of congenital color vision defects using heteroduplex-SSCP analysis. Jpn J Ophthalmol 1996 40 79 85 8739504

[23] Yang M Guo X Liu X Shen H Jia X Xiao X Li S Fang S Zhang Q Investigation of CYP1B1 mutations in Chinese patients with primary congenital glaucoma. Mol Vis 2009 15 432 7 19247456 PMC2647971

[24] Allan JA Docherty AJ Barker PJ Huskisson NS Reynolds JJ Murphy G Binding of gelatinases A and B to type-I collagen and other matrix components. Biochem J 1995 309 299 306 7619071 10.1042/bj3090299 PMC1135833

[25] Liu CJHY Ju JP Lu CL Chiu AW Altered transcripts expression of matrix metalloproteinases and their tissue inhibitors in tenon capsule of patients with glaucoma. J Glaucoma 2004 13 486 91 15534474 10.1097/01.ijg.0000137871.19942.14

[26] Rodríguez-Pla A Beaty TH Savino PJ Eagle RC Jr Seo P Soloski MJ Association of a nonsynonymous single-nucleotide polymorphism of matrix metalloproteinase 9 with giant cell arteritis. Arthritis Rheum 2008 58 1849 53 18512818 10.1002/art.23457

